# The impact of diabetes duration and glycemic control on ejection fraction in heart failure patients

**DOI:** 10.1007/s00592-025-02519-x

**Published:** 2025-05-07

**Authors:** Omer Dogan, Hasan Ali Barman, Ebru Serin, Abdullah Omer Ebeoglu, Adem Atici, Ridvan Turkmen, Ibrahim Temel, Isilay Kok, Omer Gok, Ipek Aydin, Pelinsu Elif Ozkan, Ali Nayir, Melike Kaya, Cem Kurt, Aylin Altun, Kursad Oz, Isil Uzunhasan, Murat Kazım Ersanli, Rasim Enar, Sait Mesut Dogan

**Affiliations:** 1 Department of Cardiology, Institute of Cardiology, Istanbul University-Cerrahpaşa, Istanbul, Turkey; 2 Department of Cardiology İstanbul, Sisli Hamidiye Etfal Education and Research Hospital, Istanbul, Turkey; 3 Faculty of Medicine, Department of Cardiology, Goztepe Training and Research Hospital, Istanbul Medeniyet University, Istanbul, Turkey; 4 Department of Cardiovascular Surgery, Institute of Cardiology, Istanbul University-Cerrahpaşa, Istanbul, Turkey

**Keywords:** Heart failure, Diabetes mellitus duration, Glycemic control, Ejection fraction

## Abstract

**Aim:**

The potential effects of diabetes duration and glycemic control on ejection fraction (EF) in patients with heart failure (HF) remain unclear. We investigated the impact of diabetes duration and glycemic control on ejection fraction (EF), alongside other risk factors, in HF patients with type 2 diabetes mellitus (T2DM).

**Materials and methods:**

This single-center retrospective study included 1265 patients who were admitted and discharged with a diagnosis of HF between January 2010 and January 2022, all of whom had a known diagnosis of T2DM prior to admission. The patients included in the study were divided into two groups: those with heart failure and reduced ejection fraction (HFrEF, EF ≤ 40%) and those with or mid-range or preserved ejection fraction (HFmrEF + HFpEF, EF > 40%).

**Results:**

Among the 1265 patients, 697 had HFrEF. The duration of diabetes was significantly longer (13 vs. 7 years, *p* < 0.001) and HbA1c levels were higher (8.4 ± 1.6% vs. 7.7 ± 1.5%, *p* < 0.001) in the HFrEF group. Multivariable analysis identified diabetes duration (OR 2.23, *p* < 0.001), hypertension (OR:6.62, *p* < 0.001), and the use of oral antidiabetic agents (OR 0.74, *p* = 0.042) as independent predictors of reduced EF. Prolonged diabetes duration was associated with a reduction in EF (AUC = 0.780, *p* < 0.001). Conversely, although glycemic control was poorer in the HFrEF group, it was not an independent predictor of EF.

**Conclusion:**

Prolonged diabetes duration significantly reduces EF, among HF patients with T2DM, independent of glycemic control and other risk factors. While poor glycemic control was more prevalent in HFrEF patients, it did not independently affect EF.

## Introduction

 Diabetes significantly increases the risk of heart failure (HF), with affected individuals facing a two- to four-fold higher risk than non-diabetics [1, 2]. HF in diabetes primarily results from ischemic heart disease (IHD), hypertension, hyperglycemia, obesity, and myocardial-related factors. Prolonged diabetes duration, aging, high body mass index (BMI), and chronic kidney disease (CKD) further contribute to HF risk [3, 4]. Notably, myocardial dysfunction can develop in diabetic patients even without IHD or hypertension due to complex pathophysiological mechanisms. HF risk factors in diabetes are categorized as cardiac and non-cardiac [5].

The prevalence of diabetes in HF patients remains consistent regardless of left ventricular ejection fraction (EF) [6, 7]. Diabetes is significantly associated with worse outcomes in HF, with the highest risk observed in heart failure with reduced ejection fraction (HFrEF) [5, 6]. Regardless of HF phenotype, patients with both HF and diabetes have a 50–90% higher cardiovascular (CV) mortality rate, including death from worsening HF, compared to those without diabetes [8].

Individuals with type 2 diabetes mellitus (T2DM) have higher lifetime risk of developing IHD, stroke, HF, atrial fibrillation, and peripheral artery diseases, as well as cardiovascular disease (CVD). Additionally, many patients with CVD remain undiagnosed T2DM [5–7]. Considering the significant impact of early-onset diabetes and CVD on prognosis, screening for diabetes in CVD patients and assessing cardiovascular risk in individuals with diabetes is of great importance for managing CV and kidney diseases [4, 9]. An important change in the new ESC guideline revision is the introduction of a CVD risk calculator (SCORE2-Diabetes Risk Score) aimed at estimating risk among individuals with type 2 diabetes who do not have established CVD, HF, or CKD. The guideline also highlights a significant relationship between the duration of diabetes and the prevalence, severity, mortality, and morbidity of CVD (HF, IHD, AF, sudden cardiac death) [5]. Previous studies have demonstrated the adverse effects of prolonged diabetes duration and poor glycemic control on myocardial function [5, 6]. However, studies that clearly elucidate the independent effects of these two factors on ejection fraction (EF) are limited. Therefore, this study evaluates the effects of diabetes duration and glycemic control on EF and investigates other potential risk factors associated with EF reduction in patients with heart failure.

## Materials and methods

In this single-center retrospective study, 1265 (47.5%) of a total of 2658 patients who were hospitalized with a diagnosis of HF, treated and discharged between January 2010 and January 2022, who met the study criteria were included.

Inclusion Criteria: (a) Patients admitted and treated for decompensated HF, who survived and were discharged. (b) Patients with known T2DM prior to the index hospitalization, with stress hyperglycemia ruled out according to relevant guideline criteria during hospitalization and post-hospital follow-up.

Exclusion Criteria: Patients without known diabetes at baseline or diagnosed with diabetes after the HF diagnosis were excluded. Additionally, patients who did not attend regular follow-ups, had severe organ dysfunction, metastatic malignancy, or were pregnant were excluded from the study.

Patient data were collected from electronic medical records and clinical databases. Personalized, evidence-based treatments were administered at regular intervals. Patients were classified into HF subgroups based on EF (HFrEF, HFmrEF, and HFpEF) and further analyzed in two groups (EF ≤ 40% and EF > 40%). Diabetes duration was recorded. The study complied with the Declaration of Helsinki and received Institutional Review Board (IRB) approval.

### Definitions

HF was categorized by reduced EF (HFrEF), mid-range EF (HFmrEF) and preserved EF (HFpEF). HF diagnosis was based on the Framingham score (2 major criteria or 1 major and 2 minor criteria) and evidence of systolic or diastolic dysfunction on Doppler echocardiograph. Patients with signs or symptoms of HF and a left ventricular (LV) ejection fraction (EF) ≤ 40% were considered to have HF with reduced EF (HFrEF). Patients with signs or symptoms of HF, LVEF between 40% and 49% were considered to have HF with mid-range EF. Patients with signs or symptoms of HF, LVEF ≥ 50%, and evidence of diastolic LV dysfunction were considered to have HF with preserved EF (HFpEF) [10]. Diastolic dysfunction was defined by the ratio of passive transmitral LV inflow velocity to tissue Doppler imaging velocity of the medial mitral annulus during passive filling (E/e’), with a ratio > 15 considered to be dysfunction. The NYHA functional classification has been used to describe the severity of symptoms and exercise intolerance [11].

Diabetes was defined as HbA1c greater than or equal to 47.5 mmol/mol (6.5%), self-reported physician-diagnosed diabetes mellitus type 2 or current use of antidiabetic medications [12]. The definition of duration of diabetes was derived from the question: “What was your age when the diabetes was first diagnosed?”. International Classification of Diseases (ICD) codes in the national database or hospital system were used for the patients’ initial diabetes diagnoses. At baseline, self-reported DM history was combined with ICD codes to identify DM.

## Transthoracic echocardiography

Data regarding transthoracic echocardiography reports were taken from studies which had been performed using a Philips iE33 echocardiography machine and X5 transducer (Philips Healthcare, Andover, MA, USA) with the patient in the left lateral decubitus position. The standard evaluation included M-mode, 2-D, and Doppler studies according to the recommendations of the American Society of Echocardiography. LV ejection fraction was calculated from apical four and two chamber views by manually tracing end-diastolic and end-systolic endocardial borders, using Simpson’s method [13].

### Statistical analysis

All statistical tests were conducted using the Statistical Package for the Social Sciences 25.0 for Windows (SPSS Inc., Chicago, IL, USA). The Kolmogorov-Smirnov test was used to analyze normality of the data. Continuous data are expressed as mean ± SD, and categorical data are expressed as percentages. Chi-square test was used to assess differences in categorical variables between groups. Student’s t-test or Mann Whitney U test was used to compare unpaired samples as needed. Univariate and multivariate logistic regression analysis were used to identify independent variables of reduced EF development. After performing univariate analysis, significantly obtained variables were selected into the multivariate logistic regression analysis with the stepwise method. The results of univariate and multivariate regression analyses were presented as odds ratio with 95% CI. For the duration of diabetes, receiver operating characteristic (ROC) curves were obtained and the optimal values with the greatest total sensitivity and specificity in the prediction of reduced EF development were selected. Significance was assumed at a 2-sided *p* < 0.05.

## Results

Table 1 outlines the demographic and clinical characteristics of the study cohort, stratified by ejection fraction. The cohort comprised 697 patients with heart failure and reduced ejection fraction (HFrEF, EF ≤ 40%) and 568 patients with preserved or mid-range ejection fraction (HFmrEF + HFpEF, EF > 40%).

**Table 1:** The demographic and clinical data of the study population

Variables	(EF ≤ 40), *n* = 697	(EF > 40), *n* = 568	*P* value
Age (years)	66.9 ± 11.5	66.1 ± 11.5	0.231
Male, n(%)	444 (63.7)	351 (61.8)	0.520
Smoker	395 (56.7)	325 (57.3)	0.820
BMI (kg/m^2^)	26.5 ± 4.1	27.2 ± 3.8	0.122
NYHA Class	2.2 ± 0.7	2.0 ± 0.7	0.016
Diabetes duration (years), mean (min-max)	13 (1–41)	7 (1–22)	< 0.001
Ejection fraction (%)	33.2 ± 4.8	50.8 ± 6.3	< 0.001
LA diameter (mm)	44.8 ± 6.0	43.8 ± 5.9	0.132
PASP (mmHg)	39.7 ± 19.9	36.9 ± 19.5	0.549
Systolic blood pressure (mmHg)	123.5 ± 19.6	127.4 ± 20.7	0.351
Diastolic blood pressure (mmHg)	75.6 ± 12.5	74.8 ± 12.6	0.267
Death, n(%)	106 (15.2)	91 (16.0)	0.682
Medical History, n(%)			
Ischemic heart disease	567 (81.3)	460 (80.9)	0.817
Atrial fibrillation	201 (28.8)	178 (31.3)	0.355
Hyperlipidemia	384 (55.1)	293 (51.6)	0.234
Hypertension	545 (78.2)	303 (53.3)	< 0.001
Chronic kidney disease	135 (19.4)	108 (19.0)	0.886
Cerebrovascular disease	81 (11.6)	72 (12.7)	0.603
COPD	80 (11.5)	65 (11.4)	0.985
Treatment			
ACEi/ARB	532 (76.4)	437 (76.9)	0.841
ARNI	66 (9.4)	45 (7.9)	0.864
Beta blocker	574 (82.4)	482 (84.9)	0.254
SGLT-2 Inhibitors	165 (23.7)	139 (24.5)	0.741
MRA	275 (39.5)	254 (44.4)	0.067
Digoxin	52 (7.5)	42 (7.4)	0.971
Oral anticoagulant	218 (31.3)	180 (31.7)	0.858
Ivabradin	27 (3.9)	12 (2.1)	0.073
Diuretics	450 (64.5)	334 (58.8)	0.046
Statin	482 (69.2)	383 (67.4)	0.512
Oral antidiabetic agent	578 (82.9)	501 (88.2)	0.046
Insulin therapy	122 (17.5)	92 (16.1)	0.415
Cardiac device therapy	69 (9.9)	47 (8.3)	0.329
Laboratory Findings			
Fasting glucose (mg/dl)	146.6 ± 51.3	142.5 ± 52.6	0.352
HbA1c (%)	8.4 ± 1.6	7.7 ± 1.5	< 0.001
eGFR	66.9 ± 15.9	68.4 ± 17.8	0.114
Total cholesterol ((mg/dl))	189.2 ± 48.3	197.3 ± 45.6	0.601
HDL cholesterol ((mg/dl))	43.5 ± 12.7	42.6 ± 12.0	0.677
Creatinine (mg/dl)	1.1 ± 0.9	1.1 ± 0.8	0.897
Uric Acide (mg/dl)	5.9 ± 2.0	5.8 ± 2.1	0.779
Sodium (mmol/l)	139.4 ± 4.1	139.1 ± 4.0	0.178
Potassium (mmol/l)	4.4 ± 0.5	4.5 ± 0.5	0.505
Albümin (g/dl)	3.7 ± 0.9	3.8 ± 0.8	0.238
Hemoglobin (g/dl)	12.5 ± 1.9	12.7 ± 2.2	0.226
Troponin (ng/dl) mean (min-max)	32 (1–42300)	23 (1–30400)	0.010
NT-ProBNP (pg/ml) mean (min-max)	1096 (63–26347)	867 (62–35000)	0.518

*HFrEF* Heart failure with reduced ejection fraction; *EF* Ejection fraction; *HFmrEF* Heart failure with mid-range EF; *HfpEF* Heart failure with preserved EF; *BMI* Body mass index; *NYHA* New York Heart Association; *LA* Left atrium; *PASP* pulmonary artery sistolic pressure; *COPD* Chronic obstructive pulmonary disease; *ACEi* Angiotensin-converting enzyme inhibitors; *ARB* Angiotensin receptor blocker; *ARNI* Angiotensin Receptor-Neprilysin Inhibitor; *SGLT-2*, Sodium-Glucose Transport Protein 2; *MRA* Mineralocorticoid receptor antagonist; *HbA1c* Glycated hemoglobin; *NT-ProBNP* N-terminus pro-B-type natriuretic peptide

The mean age of patients with HFrEF was 66.9 ± 11.5 years, compared to 66.1 ± 11.5 years for patients with HFmrEF + HFpEF, with no significant difference observed (*p* = 0.231). Male predominance was seen in both groups, with 63.7% and 61.8% males in the HFrEF and HFmrEF + HFpEF groups, respectively (*p* = 0.520). Smoking status did not significantly differ between the two groups (*p* = 0.820).

**Fig. 1:**
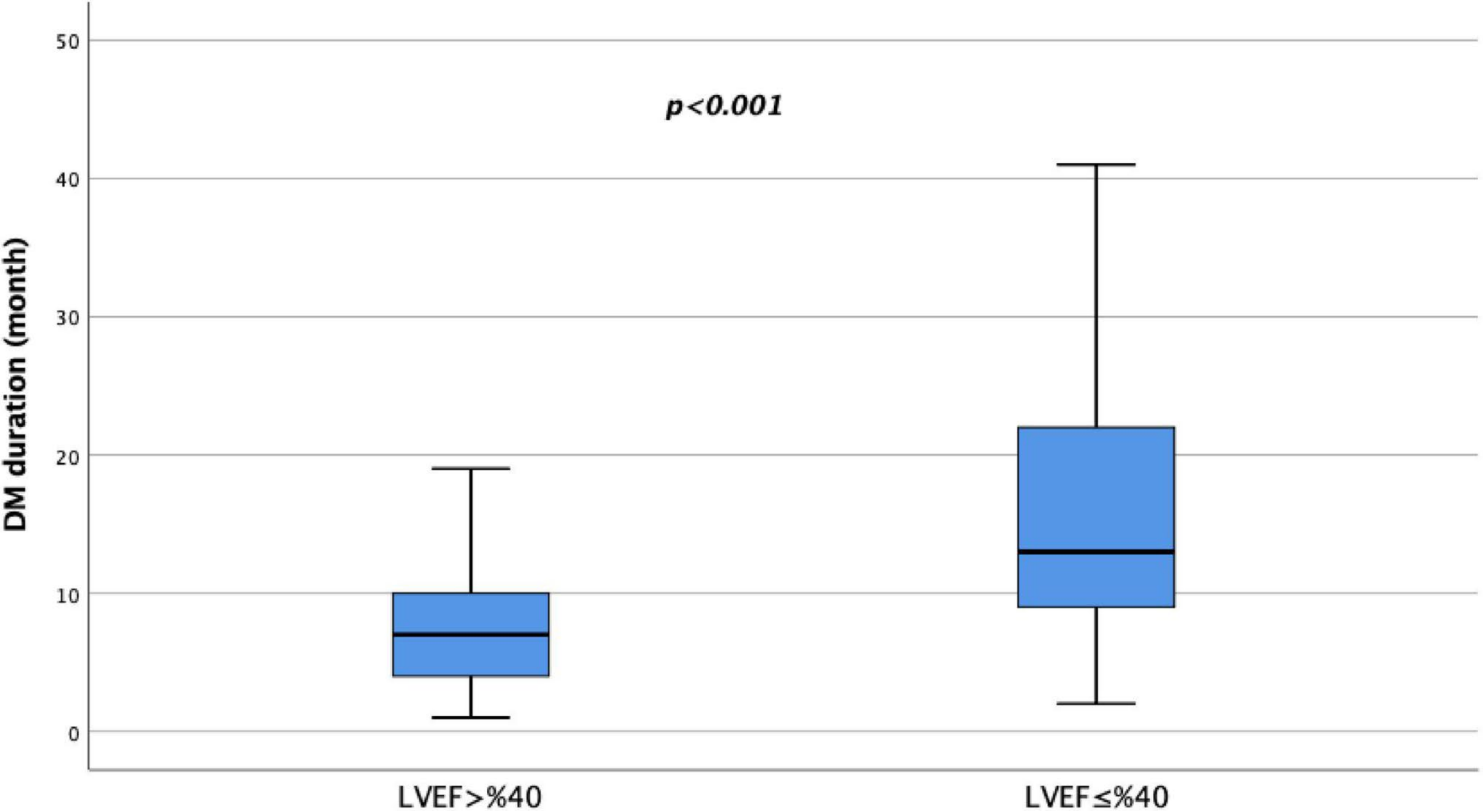
Diabetes duration according to ejection fraction

In terms of clinical variables, patients with HFrEF had a longer mean duration of diabetes compared to those with HFmrEF + HFpEF (13 vs. 7 years, *p* < 0.001) (Fig. 1). Additionally, HFrEF patients had a significantly lower mean ejection fraction (EF) compared to HFmrEF + HFpEF patients (33.2 ± 4.8% vs. 50.8 ± 6.3%, *p* < 0.001). BMI did not differ between groups (*p* = 0.122). New York Heart Association (NYHA) functional class was also significantly different between the groups, with patients in the HFmrEF + HFpEF group having a lower mean NYHA class compared to those in the HFrEF group (2.0 ± 0.7 vs. 2.2 ± 0.7, *p* = 0.016). There were no significant differences between the groups in terms of left atrial (LA) diameter, pulmonary artery systolic pressure (PASP), systolic and diastolic blood pressure and mortality rates.

Regarding medical history, hypertension was more prevalent among HFrEF patients compared to HFmrEF + HFpEF patients (78.2% vs. 53.3%, *p* < 0.001). Conversely, the prevalence of ischemic heart disease, atrial fibrillation, hyperlipidemia, chronic kidney disease, cerebrovascular disease, and chronic obstructive pulmonary disease (COPD) did not significantly differ between the groups. Regarding treatment, there were no significant differences in the utilization of angiotensin-converting enzyme inhibitors (ACEi)/angiotensin receptor blockers (ARB), angiotensin receptor-neprilysin inhibitors (ARNI), beta-blockers, sodium-glucose transport protein 2 (SGLT-2) inhibitors, mineralocorticoid receptor antagonists (MRA), digoxin, oral anticoagulants, ivabradine, or statins between the two groups. However, HFrEF patients were more likely to receive diuretics compared to HFmrEF + HFpEF patients (64.5% vs. 58.8%, *p* = 0.046). Laboratory findings revealed a significantly higher mean glycated hemoglobin (HbA1c) level in HFrEF patients compared to HFmrEF + HFpEF patients (8.4 ± 1.6% vs. 7.7 ± 1.5%, *p* < 0.001). Troponin levels were also significantly higher in HFrEF patients compared to HFmrEF + HFpEF patients (32 vs. 23 ng/dl, *p* = 0.010), whereas there were no significant differences in other laboratory variables including fasting glucose, creatinine, eGFR, total cholesterol, HDL-cholesterol, uric acid, sodium, potassium, albumin, hemoglobin, and N-terminus pro-B-type natriuretic peptide (NT-ProBNP) levels between the groups (Table 1).

Logistic regression was carried out by univariate and multivariate analyses to predict occurrence of reduced EF in heart failure patients. Age, gender, smoking status, BMI, NYHA class, diabetes duration, troponin levels, creatinine levels, hemoglobin levels, HbA1c levels, uric acid levels, pulmonary artery systolic pressure (PASP), presence of ischemic heart disease, hypertension, chronic kidney disease (CKD), atrial fibrillation, use of specific medications such as ACEi/ARB, ARNI, beta blockers, SGLT-2 inhibitors, MRA, digoxin, diuretics, oral antidiabetic agents, and insulin therapy were evaluated in univariate analysis. Variables such as NYHA class, diabetes duration, HbA1c levels, hypertension, MRA, diuretics and oral antidiabetic agents usage were re-evaluated in multivariate analysis. Diabetes duration (OR:2.23, *p* < 0.001), hypertension (OR:6.62, *p* < 0.001) and oral antidiabetic agents usage (OR:0.74, *p* = 0.042) were determined as the independent predictors of reduced EF development (Table 2).

**Table 2:** Univariate and multivariate logistic regression analyzes to identify independent predictors of reduced EF in heart failure patients

Variables Univariate Multivariate						
	OR	95%CI	*p* value	OR	95%CI	*p* value
Age	1.00	0.99–1.01	0.231			
Gender	1.08	0.86–1.36	0.485			
Smoker	1.02	0.82–1.28	0.817			
BMI	0.95	0.90–1.01	0.122			
NYHA class	1.19	1.03–1.37	0.017	1.19	0.99–1.43	0.060
Diabetes duration	2.98	1.75–6.21	< 0.001	2.23	1.20–4.27	< 0.001
HbA1c	1.30	1.21–1.39	< 0.001	1.05	0.96–1.15	0.269
Troponin	1.00	0.99*1.01	0.393			
Creatinine	1.04	0.91–1.19	0.513			
Hemoglobin	0.96	0.91–1.02	0.227			
Uric acide	1.00	0.95–1.06	0.779			
PASP	1.00	0.99–1.00	0.541			
Hypertension	3.13	2.45–4.00	< 0.001	6.62	4.81–9.11	< 0.001
Ischemic heart disease	0.96	0.77–1.21	0.772			
CKD	0.97	0.73–1.29	0.873			
Atrial fibrillation	1.12	0.88–1.43	0.334			
ACEi/ARB	1.03	0.79–1.34	0.779			
ARNI	0.94	0.49–1.79	0.864			
Beta blocker	1.20	0.88–1.62	0.233			
SGLT-2 Inhibitors	1.04	0.80–1.35	0.741			
MRA	1.24	0.99–1.55	0.059	0.81	0.61–1.07	0.143
Digoxin	0.99	0.65–1.51	0.971			
Diuretics	1.26	1.00–1.57	0.042	0.88	0.65–1.20	0.441
Oral antidiabetic	0.73	0.59–0.92	0.008	0.74	0.56–0.98	0.042
Insulin therapy	0.86	0.60–1.23	0.415			

*EF* Ejection fraction; *BMI* Body mass index; *NYHA* New York Heart Association; *HbA1c* Glycated hemoglobin; *PASP* pulmonary artery sistolic pressure; *CKD* Chronic kidney disease; *ACEi* Angiotensin-converting enzyme inhibitors; *ARB* Angiotensin receptor blocker; *ARNI* Angiotensin Receptor-Neprilysin Inhibitor; *SGLT-2* Sodium-Glucose Transport Protein 2; *MRA* Mineralocorticoid receptor antagonist

**Fig. 2:**
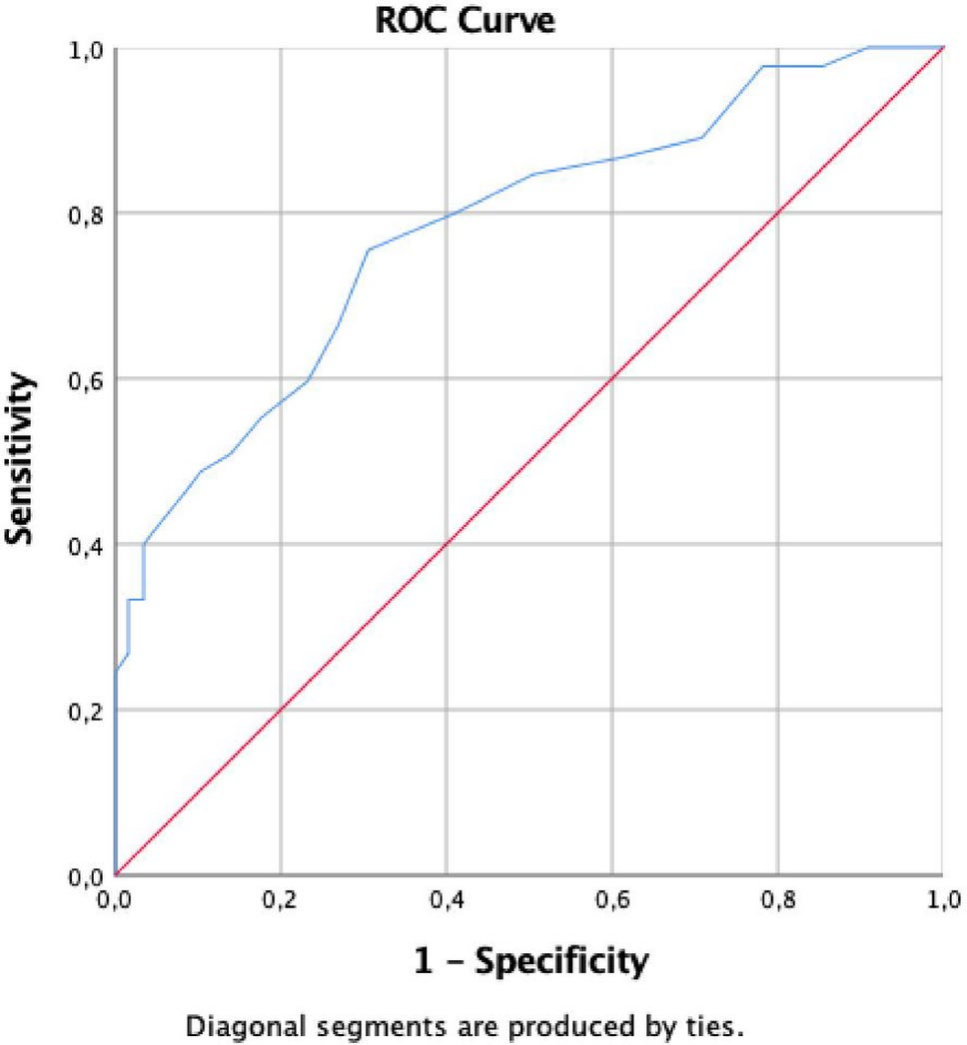
The ROC curves for diabetes duration

**Fig. 3:**
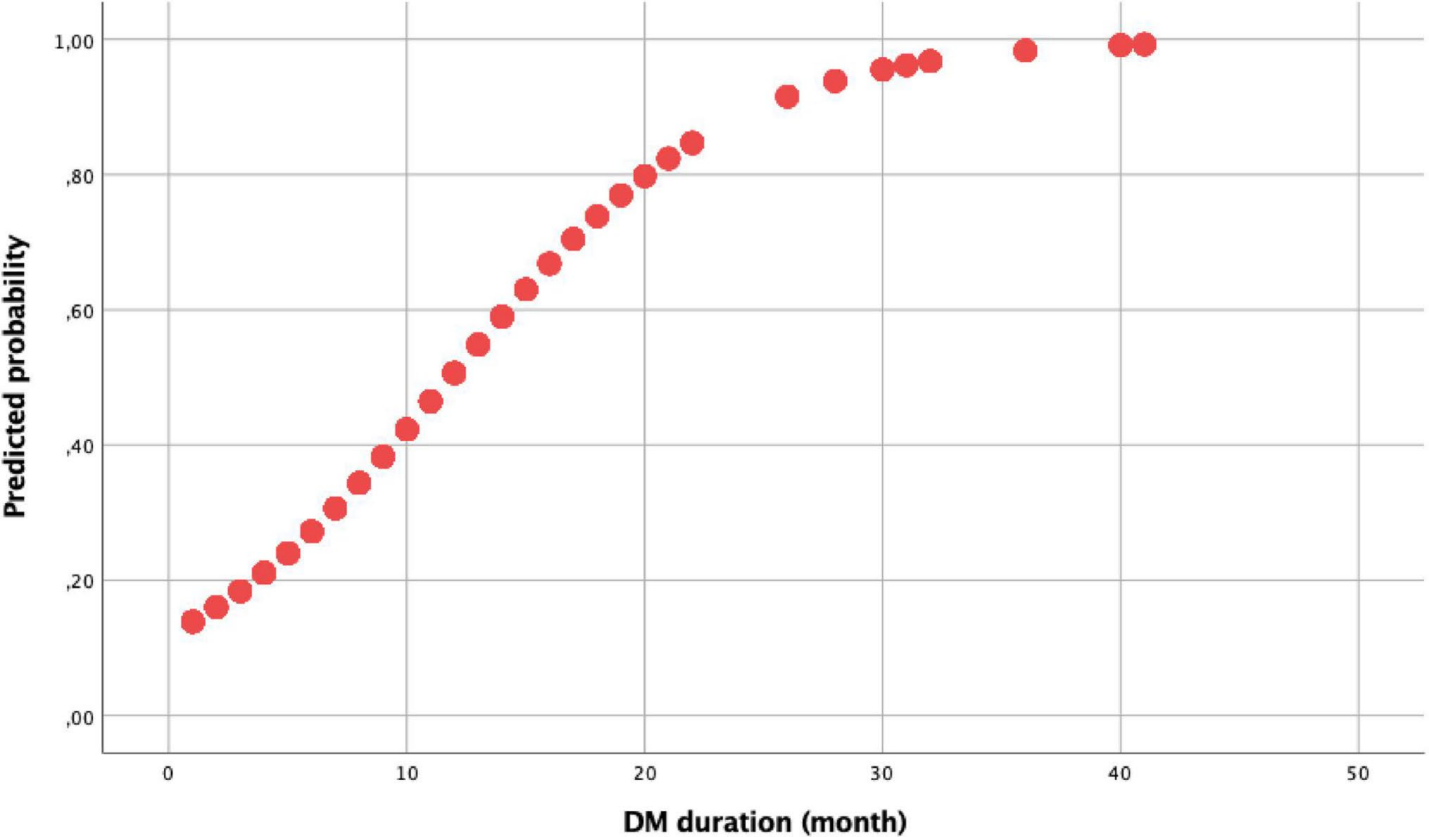
The predictive level of reduced ejection fraction group based on the duration of diabetes mellitus

The specificity and sensitivity of the diabetes duration predicting the development of reduced EF in heart failure patients were evaluated by ROC analysis (Fig. 2). The area under the curve (AUC) was measured as 0.780 (0.754–0.805), *p* < 0.001. Diabetes duration was determined to have a cut-off value of 8.5 years with 75% sensitivity and 70% specificity. In addition, the predictive value of reduced EF according to the duration of diabetes of the patients was determined by logistic regression analysis. The probability of reduced EF depending on the duration of diabetes is shown in Fig. 3.

## Discussion

This study is the first to investigate the impact of diabetes duration and glycemic control on ejection fraction among a population of heart failure (HF) patients with T2DM, in addition to other risk factors. This study demonstrates that the duration of diabetes reduces EF in patients with heart failure, underscoring the need for early diagnosis and intervention. Reducing cardiovascular risk in the management of diabetes is also of critical importance. These findings advocate for the implementation of more comprehensive cardiovascular assessment and treatment strategies in clinical practice, taking into account the long-term effects of diabetes. Moreover, the early introduction of cardioprotective treatment options in diabetic patients may contribute to the prevention of heart failure development.

Diabetes mellitus independently impairs cardiac function and increases HF risk [5]. Diabetic myocardial damage first affects diastolic function, followed by systolic dysfunction. Significant diastolic dysfunction occurs around four years after diabetes onset, regardless of coronary artery disease or hypertension. Systolic dysfunction may also develop over time [14, 15].

Prolonged diabetes duration is associated with a higher risk of heart failure (HF), independent of age, blood sugar control, and coronary heart disease, and a study on type 2 diabetes found that while both diabetes duration and age at diagnosis were linked to macrovascular events and mortality, only diabetes duration was independently related to microvascular events [5–7, 16]. Previous studies, such as the CARDIA and ARIC studies, provide valuable insights into the relationship between diabetes and heart failure. While these studies emphasize the role of HbA1c and diabetes duration, they fail to consider other factors that could further clarify the pathophysiology of heart failure in diabetic patients (17,18). Studies have shown that the duration of diabetes, alone or with other risk factors, increases the risk of heart failure (HF) [17, 18]. Recent ESC guidelines identify prolonged diabetes duration as a risk factor for HF, emphasizing integrated care, early screening, and personalized treatment to reduce cardiovascular risks. Strategies like SGLT-2 inhibitors and comprehensive cardiovascular assessments align with these recommendations, highlighting the clinical relevance of our study’s findings [5].

Poor glycemic control and longer diabetes duration are linked to increased cardiovascular disease (CVD) risk and mortality. Poor glycemic control is specifically associated with intracranial atherosclerotic stenosis, coronary heart disease (CHD), stroke, HF, and premature death [19, 20]. HF incidence rises with worsening glycemic control in diabetes mellitus (DM) patients. A study reported an 8% increase in HF risk per 1% rise in HbA1c, while another found higher HF-related hospitalization rates with poor glycemic control [20, 21]. Research in the UK suggested that poor glycemic control, alone or with factors like diabetes duration, contributes to HF development [18]. These findings indicate that inadequate glycemic control in patients with diabetes is not only a significant risk factor for cardiovascular disease (CVD) but is also directly linked to the development of heart failure and an increased rate of hospitalizations.

The lack of a significant association between HbA1c levels and EF in our study may appear to contradict the existing literature. However, this discrepancy may arise from the methodological limitations of our study and the context of the measures used. While HbA1c typically reflects glycemic control over the past 2–3 months, chronic complications of diabetes, such as microvascular changes, may show a more pronounced relationship with long-term glycemic control. The observed higher use of oral hypoglycemic agents in our study suggests that participants may have experienced poorer glycemic control in the past, which could have led to more pronounced microvascular effects over time. In this context, the inability of HbA1c to predict EF is not solely a reflection of current glycemic control, but could be related to more complex biological interactions and mechanisms. This issue remains an area that warrants further exploration through in-depth comparisons with other studies in the literature [22, 23]. Moreover, this study primarily focuses on commonly used predictors such as HbA1c and diabetes duration. However, other potential biomarkers and factors may also influence cardiac function. For instance, glycemic variability, fasting insulin levels, and inflammatory markers (e.g., CRP, IL-6) could be associated with heart failure development and the decline in EF (14,15).These factors may provide a more comprehensive understanding of the cardiovascular effects of diabetes, and further investigation into these markers is recommended in future studies.

The prolonged duration of diabetes can significantly contribute to a decline in EF in patients with heart failure through a range of interconnected mechanisms. Chronic hyperglycemia, along with the development of insulin resistance, plays a central role in the progression of endothelial dysfunction, which promotes vascular stiffness and atherosclerosis. These changes can impair coronary blood flow and myocardial perfusion, leading to myocardial ischemia. Over time, this ischemia causes left ventricular remodeling, which ultimately reduces cardiac output and contributes to a decline in EF. In addition to these vascular changes, prolonged hyperglycemia results in the accumulation of advanced glycation end-products (AGEs) in myocardial tissue. AGEs have been shown to promote fibrosis, which not only impairs myocardial contractility but also reduces myocardial compliance, further contributing to heart failure progression. Furthermore, diabetes induces significant metabolic disturbances, including altered lipid metabolism, which can exacerbate cardiac dysfunction. This dysregulated metabolism, coupled with chronic hyperglycemia, promotes a state of persistent inflammation and oxidative stress, which accelerates myocardial damage [24–27]. The coexistence of diabetes and heart failure establishes a detrimental cycle, where each condition aggravates the other. Diabetes exacerbates myocardial function through metabolic and structural changes, whereas heart failure impairs glucose control and metabolic regulation, ultimately resulting in a progressive decline in EF. This interaction between diabetes and heart failure underscores the critical need for early intervention and comprehensive management to mitigate the progression of both conditions and enhance patient outcomes [25–29].

Our study has several limitations. The first is that it is retrospective and single-center in nature. The second limitation is related to the assessment of glycemic control, which was based on fasting blood glucose measurements and other glycemic readings, such as HbA1c, taken during hospital admission. A more accurate estimation of glycemic control could have been achieved by considering values prior to hospitalization. As a result, we may have underestimated the true relationship between glycemic control and ejection fraction (EF). Future research should investigate the effects of glycemic variability on EF in more detail. Third, in evaluating the age at diabetes onset in our study, we attempted to address this limitation by using International Classification of Diseases (ICD) codes available in national databases or hospital systems, in addition to asking patients about the time of their diabetes diagnosis.

In conclusion, our findings indicate that prolonged diabetes duration significantly reduces EF, independent of other existing risk factors. However, poor glycemic control did not show a significant effect on EF. These results emphasize the significance of early screening and cardiovascular risk management in preventing the development of heart failure in patients with a prolonged duration of diabetes. In future studies, prospective and multicenter investigations involving patients with diverse demographic characteristics would be valuable for assessing the generalizability of the findings. Furthermore, evaluating the validity of these results in patients from different regions and ethnic groups is essential for establishing the international applicability of the study.

## Data Availability

Data supporting the findings of this study are available from the corresponding author on request.
